# MicroRNA miR-188-5p as a mediator of long non-coding RNA MALAT1 regulates cell proliferation and apoptosis in multiple myeloma

**DOI:** 10.1080/21655979.2021.1920325

**Published:** 2021-05-04

**Authors:** Hui Liu, Zuofei Chi, Hong Jin, Wei Yang

**Affiliations:** a Department of Hematology, Shengjing Hospital of China Medical University, Shenyang, China; b Department of Pediatric Hematology, Shengjing Hospital of China Medical University, Shenyang, China; c Department of Pathogenic Biology, College of Basic Medical Science, China Medical University, Shengyang, China

**Keywords:** Multiple myeloma, long non-coding RNAs, cell cycle, apoptosis, microRNAs, MALAT1

## Abstract

Multiple myeloma (MM), a malignancy of plasma cells mainly derived from the bone marrow, has remained incurable generally. LncRNA MALAT1 has been reported to be upregulated in the MM cells and knockdown of MALAT1 inhibited MM cell cycle progression and enhanced cell apoptosis. Online target prediction showed that two target sites for MALAT1 existed in miR-188-5p, which has been identified as a tumor suppressor in other types of cancers. However, the role of miR-188-5p in the MM and whether miR-188-5p mediates the MM tumor progression regulated by MALAT1 are still unknown. Herein, four main MM cell lines were adopted to investigate the effects of miR-188-5p on cell proliferation and apoptosis via transfection with miR-188-5p mimic/inhibitor and co-transfection with miR-188-5p inhibitor and MALAT1-shRNA plasmids. Xenograft tumor model was also established to study these effects in vivo. Overexpression of miR-188-5p inhibited cell viability, cell proliferation as well as tumor growth and arrested cell cycle at G1 to S transition, but miR-188-5p knockdown showed opposite effects on the MM cells in vitro and in vivo. Moreover, MALAT1 was shown to be inversely correlated with miR-188-5p expression through direct binding to miR-188-5p, and in turn, miR-188-5p could mediate the MM cell proliferation and apoptosis regulated by MALAT1. These findings indicate that miR-188-5p serves as a tumor suppressor in the progression of the MM and is directly involved in MM cell proliferation and apoptosis regulated by MALAT1, which may provide a potential therapeutic target or prognostic indictor for MM clinical treatment.

## Graphical abstract

**Figure:**
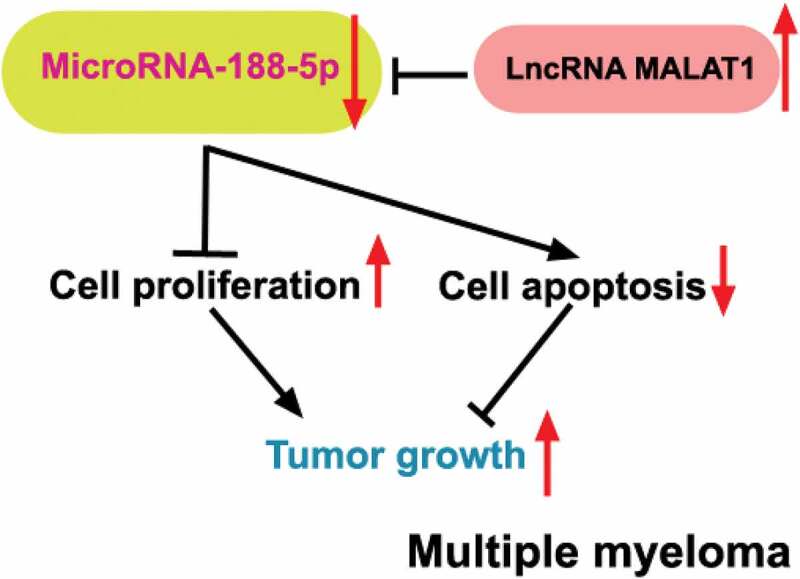

## Background

Although multiple myeloma (MM) is a relatively rare cancer that derives from abnormal plasma cells, it still represents around 10% of all blood malignancies [1,2]. It has been studied that the occurrence of MM was closely linked to the age (diagnosed patients were mostly over 61), sex (more men than women) and race (more black than white), but the reason behind this is less investigated [3,4]. Some common symptoms of MM patients are defined as CRAB including hypercalcemia, renal failure, anemia as well as bone lesions, which are also regarded as parts of diagnostic criteria of the MM worldwide [1,5]. For decades, numerous novel drugs have been introduced and applied for MM clinical therapy [2]. However, even though the overall survival of MM patients has been improved remarkably, this cancer remains incurable [2,6,7]. Hence, it becomes necessary to further explore new therapeutic strategies of MM to ameliorate patient prognosis.

Single-stranded microRNAs (miRNAs) are small endogenous non-coding RNA molecules (22–25 nt), which mainly affect various biological processes at a post-transcriptional manner [8]. Increasing researches have revealed that dysregulation of multiple miRNAs are highly related to the occurrence and development of cancers [9,10], including the MM. Several tumor suppressor miRNAs have been found to be inactivated through hypermethylation in the MM, such as miR-203, miR-34b/c, and also miR-188-5p [11–13]. It has already been verified that miR-188-5p not only inhibited gastric cancer development [14], but also suppressed hepatocellular carcinoma and prostate cancer progression [15,16]. Meanwhile, miR-188 has been found to restrain cell proliferation and cell cycle transition from G1 to S transition in the glioma and nasopharyngeal cancer cells [17,18]. Specifically, in nasopharyngeal carcinoma cells, miR-188 could arrest cell cycle progression through direct binding and targeting several cyclin-related regulators including cyclinD1 and cyclinE1 [18]. Besides, miR-188 was found to inhibit retinoblastoma protein (Rb) phosphorylation and thereby E2F activities, both of which are crucial for DNA replication and cell proliferation [18]. However, the roles of miR-188 in the proliferation and cell cycle distribution of the MM cells have not been studied yet.

Endogenous long non-coding RNAs (lncRNAs) can act as a sponge of miRNAs via binding to miRNAs through their miRNA recognition elements (MREs) and thereby inhibit the effects of miRNAs and affect relevant mRNA expression. LncRNAs (~200 nt) have been well-studied to be involved in various biological processes ranging from chromatin modification, transcriptional regulation to post-transcriptional regulation [19]. Recently, lncRNA metastasis-associated lung adenocarcinoma transcript 1 (MALAT1) has been published to be overexpressed in the MM cells and knockdown of MALAT1 suppressed MM cell proliferation and resulted in cell cycle arrest and apoptosis [20]. However, it is unclear which molecules engage in the functions of MALAT1 in cell proliferation and apoptosis during MM development.

According to the online prediction tool, MALAT1 was shown to contain two miR-188-5p binding sites. Combined with the negative regulation of miR-188-5p in the progression of other cancers, here, we hypothesized a possible mechanism that miR-188-5p may be involved in the MM cell proliferation and development regulated by MALAT1. Therefore, the present study mainly focused on the influence of miR-188-5p on MM cell proliferation and apoptosis and verified whether miR-188-5p mediated the function of MALATA1 in MM cell development.

## Methods

### Cell culture

Four human MM cell lines RPMI-8226, U266, MM.1S and NCI-H929 were utilized in this study. RPMI-8226 cells were obtained from the Shanghai Cell Bank, Chinese Academic of Sciences (China). U266 and MM.1S cells were acquired from the American Type Culture Collection (ATCC, Manassas, VA, USA). NCI-H929 cells were obtained from the Shanghai Zhong Qiao Xin Zhou Biotechnology Co., Ltd. (Shanghai, China). All cells were cultured in RPMI-1640 medium (Gibco, Grand Island, NY, USA) with 10% fetal bovine serum (FBS, Biological Industries, Beit Haemek, Israel) and maintained at a constant temperature (37°C) with 5% CO_2_. For dual luciferase assay, 293 T cells (Shanghai Zhong Qiao Xin Zhou Biotechnology Co., Ltd.) were used and cultured in the Dulbecco’s Modified Eagle’s Medium (DMEM, Logan, Utah, USA) with 10% FBS at 37°C, 5% CO_2_.

### Cell transfection

For miRNA functional investigation, miR-188-5p mimic with NC mimic (JTS Scientific, Wuhan, China) and miR-188-5p inhibitor with NC inhibitor (JTS Scientific) were transfected into U266 and NCI-H929 cells, respectively. Lipofectamine RNAiMAX transfection reagent (Invitrogen, Carlsbad, CA, USA) was adopted for cell transfection according to the manufacturer protocol. To study the effects of miR-188-5p (NR_029708) on the function of lncRNA MALAT1 in the MM cells, transfection was performed using Lipofectamine 3000 reagent (Invitrogen) to convey pRNAH1.1 vector (GenScript, Nanjing, China) containing lncRNA MALAT1-shRNA-1 or 2 (sh-MALAT1-1 or sh-MALAT1-2) or its control (sh-NC) into RPMI-8226 and U266 cells according to the manufacturer protocol. The target sequence of sh-MALAT1-1 was 5′-GGTGGTGGTATTTAGATAA-3′ and sh-MALAT1-2 was 5′-GCGTCATTTAAAGCCTAGT-3′. Co-transfection of sh-MALAT1-1 vectors and miR-188-5p inhibitor (JTS Scientific) into RPMI-8226 and U266 cells was also performed using Lipofectamine 3000 reagent (Invitrogen) according to the manufacturer instruction. Cells after transfection were cultured at 37°C with 5% CO_2_.

### EdU staining

EdU staining was performed using kFluor647-EdU cell proliferation assay kit (Keygen Biotech, Nanjing, China). Forty-eight h after transfection, cells were maintained in 10 μM EdU (Keygen Biotech) for 12 h after PBS washing. Then, fixation and penetration of cells were performed, followed by cell incubation in Click-iT reaction mixture (Keygen Biotech). Hoechst 33,342 solution (Keygen Biotech) was used for DNA counter-staining. After mounting, the slices were observed and pictured under a microscope (Olympus, Tokyo, Japan) at 400x magnification.

### Methyl thiazolyl tetrazolium (MTT) assay

Cells (5 × 10^3^ cells per well) were seeded in the 96-well-plates. Transfections were performed after cell adherence. Detection of cell viability in U266 and NCI-H929 cells was carried out 0, 24, 48, 72, 96 h after transfection, while in co-transfected RPMI-8226 and U266 cells was measured 48 h after co-transfection. For MTT detection, cells were incubated with MTT mixture (0.5 mg/ml) (Sigma-Aldrich, Darmstadt, Germany) for 4 h at 37°C, 5% CO_2_, followed by replacing the medium with 150 μl DMSO per well (Keygen Biotech). After placing in dark for 10 min, optical density (OD) values at 570 nm were measured via a microplate reader (BIOTEK, Winooski, VT, USA).

### Flow cytometry for cell cycle and cell apoptosis detection

Cell cycle analysis kit (Beyotime, Shanghai, China) was adopted to check cell cycle distribution of samples through Propidium Iodide (PI) staining. Cells were collected by centrifugation at 310 × g for 5 min followed by fixation with ice-cold 70% ethanol for 2 h at 4°C. The cells were resuspended with 500 μl staining buffer solution (Beyotime) and then incubated with 25 μl PI (Beyotime) and 10 μl RNase A solution (Beyotime) in dark for 30 min at 37°C. Annexin V-FITC apoptosis detection kit (Beyotime) was utilized to check cell apoptosis of samples by double-staining of Annexin V-FITC and PI. Cells were collected by centrifugation at 1000 × g for 5 min followed by resuspension with 195 µl Annexin V-FITC binding solution (Beyotime). Then, the cells were incubated with 5 μl AnnexinV-FITC and 10 μl PI at room temperature for 15 min. Finally, all cell samples were detected using NovoCyte Flow Cytometers (Acea Biosciences, San Diego, CA, USA) and analyzed using the NovoExpress 1.2.5 software (ACEA Bioscience). The apoptosis rate of cells was calculated as follows: apoptosis rate = (early apoptosis (Annexin V^+^ and PI^−^) cells + late apoptosis (Annexin V^+^ and PI^+^) cells)/total cells × 100%.

### Real-time PCR

Real-time PCR was performed to measure relative expression levels of miR-188-5p. High purity total RNA rapid extraction kit (BioTeke, Beijing, China) was adopted to extract total RNA of samples. Extracted RNAs were reversely transcribed into cDNAs using M-MLV reverse transcriptase (Takara, Beijing, China) with RNase inhibitor (Takara) by Stem loop method. Followed real-time PCR reaction was performed with the help of Taq HS Perfect Mix (Takara) and SYBR Green (BioTeke). The U19 and β-actin were adopted as an internal reference. The primers were synthesized by GenScript (Nanjing, China) and sequences of them were listed as follow: miR-188-5p (forward): 5′-CGATATTCATCCCTTGCATGGT-3′; (reverse): 5′-TGCAGGGTCCGAGGTATT-3′; U19 (forward): 5′-TGGAGTTGATCCTAGTCTGG-3′; (reverse): 5′-GTGCAGGGTCCGAGGTAT-3′. MALAT1 (forward): 5′-ATACCTAACCAGGCATAACA-3′; (reverse): 5′-AGTAGACCAACTAAGCGAAT-3′; β-actin (forward): 5′-GGCACCCAGCACAATGAA-3′; (reverse): 5′-TAGAAGCATTTGCGGTGG-3′. Finally, the results were analyzed using 2^−ΔΔCT^ methods.

### Western blot (WB) analysis

Total protein extraction was performed using RIPA Lysis buffer (Beyotime) with Phenylmethanesulfonyl fluoride (PMSF, Beyotime) and then Enhanced BCA protein assay kit (Beyotime) was adopted to measure concentrations of protein samples. A certain amount of samples (15–30 μg) was loaded to undergo SDS-PAGE. The proteins were then transferred onto PVDF membranes (Thermo Fisher Scientific) through a ‘sandwich’ structure and the membranes would be incubated with diluted primary antibodies at 4°C overnight after blocking in 5% (M/V) BSA (Biosharp, Hefei, China). Then, following washing with TBST, incubation of corresponding HRP-labeled secondary antibodies was performed at 37°C for 40 min. The protein bands on the membranes were finally scanned and analyzed through Gel-Pro-Analyzer software, after incubation with ECL reagent (7-sea Biotech, Shanghai, China) for chemiluminiscence. All primary antibodies was shown as follow: cyclinD1 (1:1000, Cell Signaling Technology (CST), Danvers, MA, USA), cyclinE1 (1:1000, Proteintech, Wuhan, China), E2F1 (1:500, Proteintech), p-Rb^Ser811^ (1:400, Abclonal, Wuhan, China), p-Rb^Ser780^ (1:500, abcam, Cambridge, MA, USA), Rb (1:1000, Abclonal), cleaved-caspase-3 (1:1000, CST), cleaved-caspase-9 (1:1000, CST), Bcl-2 (1:500, Proteintech), Bax (1:500, Proteintech) and β-actin (1:2000, Proteintech). For secondary antibodies, Goat Anti-Mouse IgG-HRP (1:10,000, Proteintech) was used for β-actin and Goat Anti-Rabbit IgG-HRP (1:10,000, Proteintech) was adopted for others. Meanwhile, β-actin was treated as an internal control.

### Terminal deoxynucleotidyl transferase-mediated dUTP nick-end labeling (TUNEL) staining

Fixed cell samples were treated with 0.1% TritonX-100 (Beyotime), followed by incubating with prepared TUNEL detective solution based on the One Step TUNEL Apoptosis Assay Kit (Beyotime). After counter-staining by DAPI (Beyotime), cells were added with mounting medium (Solarbio) and finally checked and pictured under a microscope (400x) (Olympus). Tumor tissues were embedded into paraffin and cut into 5 μm slices. After de-waxing and rehydrating, TritonX-100 (0.1%, Beyotime) was used for permeabilization of slices, followed by PBS washing and TUNEL reaction through an In Situ Cell Death Detection Kit (Roche, Switzerland). Diaminobenzidine (DAB, Solarbio) and hematoxylin (Solarbio) were added to slices for coloration and counter-staining, respectively. The slices were observed under a microscope (Olympus) at a 400x magnification after mounting.

### Animal experiments

Xenograft tumor model was established using six-week-old healthy nude mice (Beijing HFK Bioscience Co., LTD, China). Before injection, nude mice were adaptively fed in a 12 h white/dark cycle with free access to water and food for one week (22 ± 1°C, 45–55% humidity). All mice were subsequently divided into three groups: Control, NC agomir and miR-188-5p agomir. The U266 cells were adopted to subcutaneously inoculate into the right armpit of all nude mice (1 × 10^6^ cells per mouse). Nine d after inoculation, tumor nodules were observed in each group. Afterward, mice in NC agomir and miR-188-5p agomir groups were injected with 1 nmol NC agomir and miR-188-5p agomir (GenePharma, Shanghai, China), respectively, into the tumors. Tumor volumes were then measured every 3 d through a caliper and after 24 d, all the nude mice were killed. Following measuring tumor weights, all the tumors were fixed and cryopreserved. All the procedures in animal experiments followed the guide for the care and use of laboratory animals and this study was approved by Shengjing Hospital of China Medical University.

### Immunohistochemistry (IHC)

Paraffin sections (5 μm) were de-waxed through xylene and rehydrated through ethanol, followed by antigen retrieval via sodium citrate. Following 3% H_2_O_2_ incubation for 15 min, sections were blocked using normal goat serum (Solarbio, Beijing, China). The primary antibody of Ki67 (abcam) was diluted in 1:500 and used to incubate sections in a wet box at 4°C overnight. Same dilution of secondary HRP-labeled Goat Anti-Rabbit IgG antibody (Thermofisher, Rockford, USA) was applied to subsequently incubate sections in a wet box at 37°C for 60 min and then DAB (Solarbio) was used for coloration. The sections were counter-stained by hematoxylin (Solarbio). After mounting, all samples were observed under a 400x microscope (Olympus).

### Bioinformatics analysis

In the present study, Starbase (http://starbase.sysu.edu.cn/) was used to predict binding sites between MALAT1 and miR-188-5p.

### Dual-luciferase reporter assay

Dual-luciferase reporter assay was performed using 293 T cells. After cell counting and seeding into 12-well plates, starvation treatment of cells was carried out when cell confluence was around 70% before transfection. Co-transfection of cells was performed using Lipofectamine 3000 reagent (Invitrogen) according to manufacturer protocol through a mixture of luciferase vector pmirGLO (Promega, Madison, Wisconsin, USA) containing predicted wildtype or mutant binding sequences (wt-MALAT1-seed1/2 or mut-MALAT1-seed1/2) or combined sequences (wt-MALAT1-seed1 + 2 or mut-MALAT1-seed1 + 2) of MALAT1 and nucleic acid fragments (miR-188-5p mimic or NC mimic, JTS Scientific). Followed detection for luciferase activity was performed 48 h after co-transfection using Dual Luciferase Reporter Assay Kit (Promega) according to the manufacturer instruction.

### Statistical analysis

All data were exhibited as mean ± standard deviation (SD). Cell experiments were performed at least three times, while animal or tumor tissue experiments contained six replicates in each group. One-way and two-way ANOVA were adopted to analyze differences among three or more groups through GraphPad Prism 8 (GraphPad Software, San Diego, CA, USA). Meanwhile, P < 0.05 was considered in this study to be statistically significant.

## Results

### miR-188-5p inhibits MM cell proliferation

**Figure 1.**
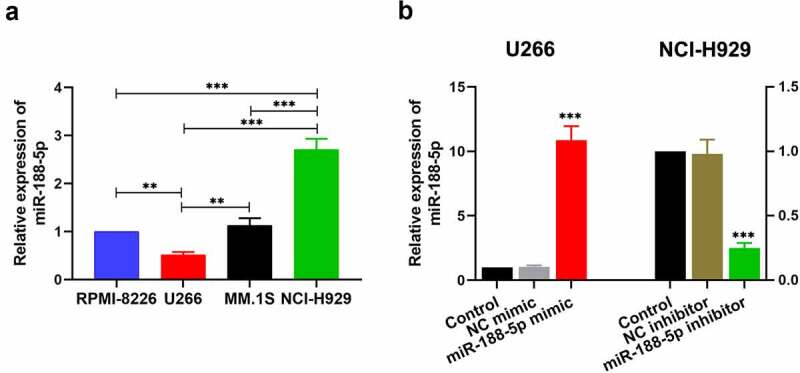
MiR-188-5p expression in the MM cells. (a). The level of miR-188-5p within four MM cell lines was measured using Real-time PCR. U266 cells and NCI-H929 cells were selected for further experiments. **P < 0.01, ***P < 0.001. (b). U266 cells were transfected with miR-188-5p mimic (and NC mimic) and NCI-H929 cells with miR-188-5p inhibitor (and NC inhibitor). Real-time PCR was performed to confirm the transfection effects. ***P < 0.001 compared with the NC mimic or inhibitor group

To explore the role of miR-188-5p in the progression of the MM, the expression of miR-188-5p was detected by real-time PCR in multiple MM cell lines, including RPMI-8266, U266, MM.1S and NCI-H929. It was notable that endogenous miR-188-5p expression in U266 cells was significantly lower than that in other cell lines (Figure 1(a)). Meanwhile, endogenous miR-188-5p expression in NCI-H929 cells was markedly higher than those in other cell lines (Figure 1(a)). Therefore, U266 and NCI-H929 cells were selected to perform further study. U266 cells with a relatively low endogenous miR-188-5p expression were applied to be transfected with miR-188-5p mimic, while NCI-H929 cells with a relatively high endogenous miR-188-5p expression were adopted for the transfection of miR-188-5p inhibitor. Their relative expression of miR-188-5p after transfection were measured by real-time PCR (Figure 1(b)), both of which showed efficient transfection effects.

**Figure 2.**
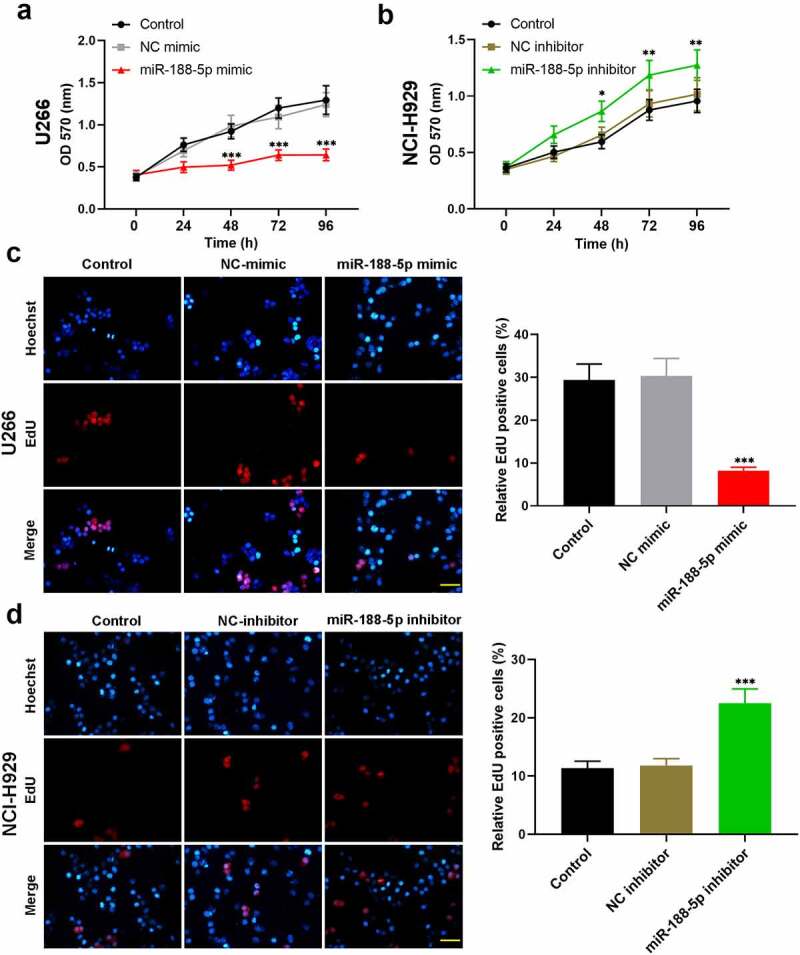
MiR-188-5p inhibits MM cell proliferation. (a and b). Cell viability at 0, 24, 48, 72, 96 h after transfection was detected using MTT assay. (c and d). EdU staining (red) was performed to label proliferating cells at 48 h after transfection. Cells were counter-stained by Hoechst 33,342 (blue). Scale bar = 50 μm. *P < 0.05, **P < 0.01 and ***P < 0.001 compared with the NC mimic or inhibitor group

**Figure 3.**
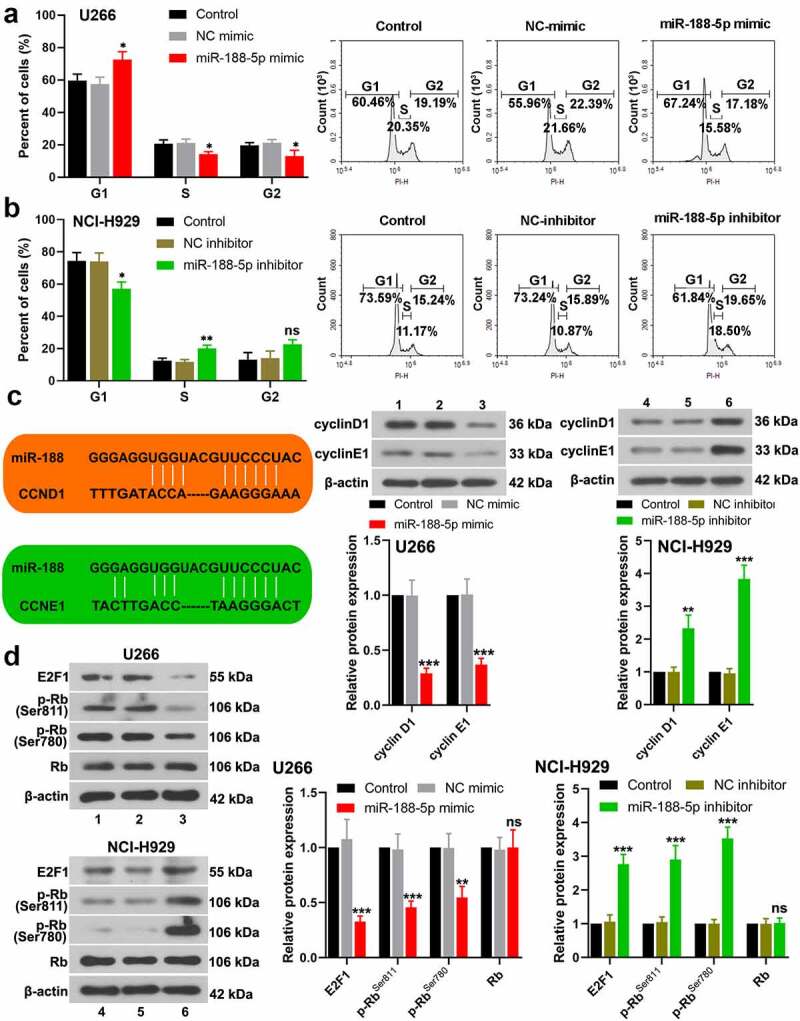
MiR-188-5p suppresses G1/S transition during MM cell cycle. (a and b). Cell cycle distribution was detected at 48 h after transfection via flow cytometry. (c). The protein levels of cyclinD1 and cyclinE1 in the MM cells were evaluated at 48 h after transfection using WB analysis. (d). The protein levels of E2F1, phosphorylated Rb at Ser811 and Ser780 and Rb in the MM cells were evaluated at 48 h after transfection using WB analysis. *P < 0.05, **P < 0.01 and ***P < 0.001 compared with the NC mimic or inhibitor group. ns: no statistical significance. 1, 2 and 3: control, NC mimic and miR-188-5p mimic. 4, 5 and 6: control, NC inhibitor and miR-188-5p inhibitor

To explore the role of miR-188-5p in MM cell viability, MTT assay was performed at 0, 24, 48, 72, and 96 h after transfection in these two cell lines. The results showed that overexpressed miR-188-5p significantly decreased the viability but knockdown of miR-188-5p obviously increased the viability of U266 cells and NCI-H929 cells (Figure 2(a and b)), S1a & b). EdU staining was performed at 48 h after transfection to label proliferating cells (cells in S phase of cell cycle progression). The quantification analysis of Edu staining verified that miR-188-5p mimic led to reductions in S phase cells but miR-188-5p inhibitor significantly increased the cells in the S phase (Figure 2(c and d)), indicating that miR-188-5p suppressed DNA replication during cell growth. Meanwhile, flow cytometry was performed to analyze the alterations of cell cycle distribution in two MM cell lines 48 h after transfection, which showed that miR-188-5p upregulation arrested cell cycle at G1 to S transition and stayed more cells in the G1 phase, but miR-188-5p knockdown showed reversed results and promoted G1/S transition during cell cycle (Figure 3(a and b)). Furthermore, the expression of other cell cycle- and DNA replication-related proteins, including cyclinD1, cyclinE1, E2F1, p-Rb^Ser811^ and p-Rb^Ser780^, was detected by WB assay. The results showed that miR-188-5p mimic decreased dramatically the protein levels of cyclinD1, cyclinE1, E2F1, p-Rb^Ser811^ and p-Rb^Ser780^, yet miR-188-5p inhibitor significantly elevated the expression levels of those proteins that are all the indicators of enhanced cell cycle progression and DNA replication (Figure 3(c and d)). Therefore, these findings suggest that miR-188-5p suppresses MM cell proliferation through inhibiting DNA replication and arresting the MM cells at G1/S transition.

### miR-188-5p enhances MM cell apoptosis

**Figure 4.**
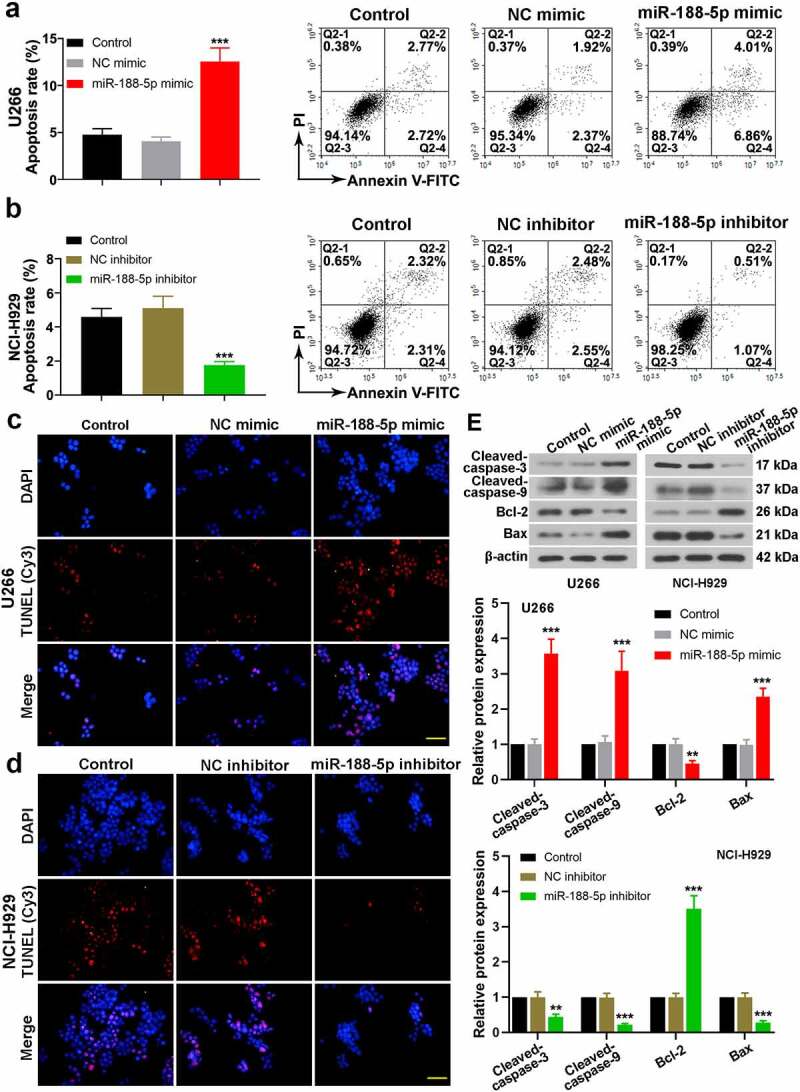
MiR-188-5p enhances MM cell apoptosis. (a and b). Flow cytometry was utilized to detect cell apoptosis via Annexin V-FITC and PI double staining. (c and d). Immunofluorescence for TUNEL (Cy3, red) and DAPI (blue) staining was performed detect cell apoptosis. Scale bar = 50 μm. (e). WB analysis of cleaved-caspase-3, cleaved-caspase-9, Bcl-2 and Bax. All experiments were performed 48 h after transfection. **P < 0.01, ***P < 0.001 compared with the NC mimic or inhibitor group

To investigate the roles of miR-188-5p on cell apoptosis in the MM, double staining of Annexin V-FITC and Propidium Iodide (PI) was performed to detect apoptotic cells via flow cytometry at 48 h after transfection. It was found that overexpressed miR-188-5p increased dramatically the apoptotic rate of U266 cells, while in NCI-H929 cells, miR-188-5p knockdown decreased cell apoptosis (Figure 4(a and b)). Besides, TUNEL staining was also employed to detect apoptotic cells, which exhibited consistent results with that in the flow cytometry (Figure 4(c and d)). Moreover, the expression of apoptosis-related proteins was measured by WB analysis (Figure 4(e)). Cleaved caspase-3 and 9 levels were markedly elevated in the U266 cells transfected with miR-188-5p mimic accompanied with reduced expression of Bcl-2 and enhanced expression of Bax, while as expected, downregulated miR-188-5p showed opposite effects on the expression of those proteins. Hence, these findings demonstrate that miR-188-5p is able to increase cell apoptosis in the MM.

### Effects of miR-188-5p on MM cell development in vivo

**Figure 5.**
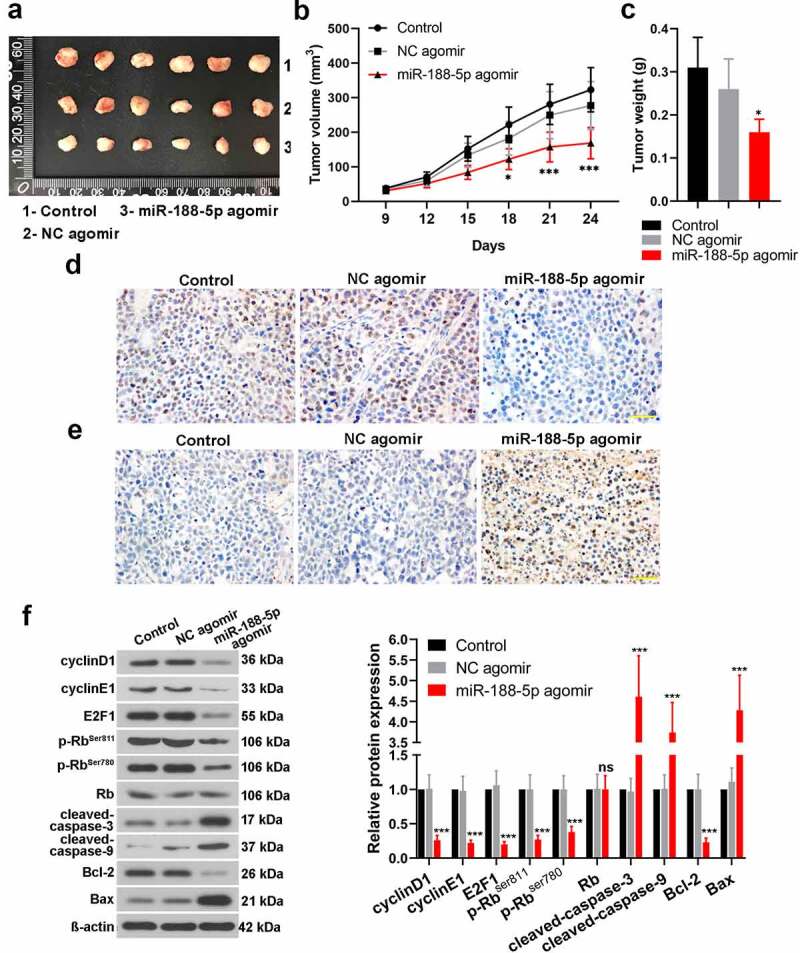
In vivo study verifies the effects of miR-188-5p on MM cell development. (a). Xenograft tumor model of MM was established through U266 cell injection with further miR-188-5p agomir (NC agomir). Tumors were taken photos 24 d after injection. 1, 2 and 3: control, NC agomir and miR-188-5p agomir. (b and c). Tumor volumes (every 3 d) and final weights were measured and analyzed. (d). Ki67 expression was detected using IHC staining (Ki67-positive cells, brown). (e). TUNEL assay was performed to detect cell apoptosis in MM tissues (apoptotic cells, brown). (f). WB was used to detect relative protein levels of cyclinD1, cyclinE1, E2F1, phosphorylated Rb at Ser811 and Ser780, Rb, cleaved-caspase-3 and 9, Bcl-2 and Bax. N = 6 mice per group. Scale bar = 50 μm. *P < 0.05 and ***P < 0.001 compared with the NC agomir group. ns: no statistical significance

To investigate the effects of miR-188-5p on the MM cells in vivo, xenograft tumor model was established. U266 cells were inoculated into the nude mice with miR-188-5p agomir (NC agomir). It was shown that miR-188-5p agomir induced smaller tumors as compared with the control and NC tumors (Figure 5(a)). Further analysis of tumor volumes and weight verified a decreased volume of tumor and a lighter tumor weight in the miR-188-5p agomir group in contrast to the NC agomir group (Figure 5(b and c)). Besides, IHC for Ki67 detection showed less Ki67-positive cells (brown) in the miR-188-5p agomir tumor tissues which indicated that upregulated miR-188-5p induced less proliferative cells in the MM (Figure 5(d)). TUNEL detection for apoptosis exhibited that miR-188-5p agomir markedly increased apoptotic cells in the MM tumors compared with the NC agomir (Figure 5(e)). WB assay analyzed the relative expressions of some cell cycle- and apoptosis-related proteins in the tumor tissues. Compared with the NC, miR-188-5p upregulation significantly reduced the expressions of cell cycle-related proteins but enhanced those of apoptosis-related proteins (figure 5(f)), which implied that miR-188-5p upregulation could inhibit cell cycle progression and induce more cell apoptosis in the MM. These results in vivo further verified that miR-188-5p plays a role in suppressing tumor growth and cell proliferation but enhancing apoptosis in the MM.

### LncRNA MALAT1 negatively regulates miR-188-5p through direct targeting

**Figure 6.**
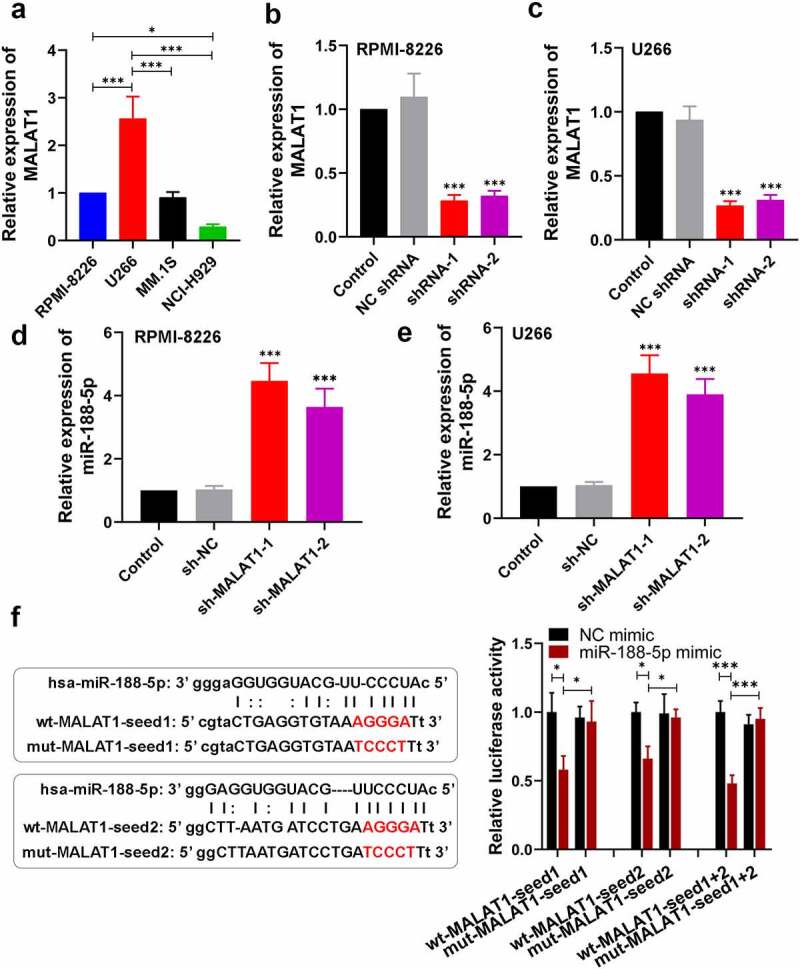
MALAT1 regulates miR-188-5p negatively through directly targeting. (a). The level of MALAT1 within four MM cell lines was measured using Real-time PCR. U266 cells and RPMI-8226 cells were selected for further experiments. *P < 0.05, ***P < 0.001. (b–e). The relative expressions of MALAT1 and miR-188-5p in RPMI-8226 and U266 cells transfected with MALAT1 shRNA-1 or 2 with its negative control (sh-MALAT1-1, sh-MALAT1-2 and sh-NC) were detected using real-time PCR. ***P < 0.001 compared with the sh-NC group. (f). Sequences of two predicted miR-188-5p binding sites on MALAT1 (seed1 and 2) were shown. Mutant sequences were marked in red. Luciferase vectors containing wildtype or mutant MALAT1-seed sequences were co-transfected with miR-188-5p mimic or NC mimic into 293 T cells. Relative luciferase activities were then determined 48 h after transfection. *P < 0.05, ***P < 0.001

To explore the relationship between MALAT1 and miR-188-5p in the progression of the MM, the expression of MALAT1 was detected by real-time PCR in multiple MM cell lines, including RPMI-8266, U266, MM.1S and NCI-H929. The RPMI-8266 and U266 cell lines showed relatively high expression of MALAT1 compared with the MM.1S and NCI-H929 cell lines (Figure 6(a)). In addition, the correlation between MALAT1 and miR-188-5p was analyzed using Pearson correlation. The results revealed that MALAT1 expressions in the MM cells were negatively correlated with miR-188-5p expressions (Figure S2; R = −0.7842, P = 0.0025). Subsequently, MALAT1 expression was silenced in the MM cells including RPMI-8226 and U266 through transfection with sh-MALAT1-1 and sh-MALAT1-2, which was confirmed by real-time PCR (Figure 6(b and c)). MiR-188-5p expression levels were detected using real-time PCR, which showed that MALAT1 knockdown significantly increased the miR-188-5p levels in the MM cells (Figure 6(d and e)). Meanwhile, two binding sites of miR-188-5p on MALAT1 were predicted using the starBase (Figure 6(f)). To verify that, dual-luciferase reporter assay was performed. Results showed remarkably lower luciferase activities in the co-transfected cells with miR-188-5p mimic and wt-MALAT1, especially wt-MALAT1-seed1 + 2 (Figure 6(f)), indicating that MALAT1 directly bound to miR-188-5p. Therefore, these findings suggest that MALAT1 directly targets miR-188-5p to negatively regulate it.

### MiR-188-5p involves in MM cell development regulated by MALAT1

**Figure 7.**
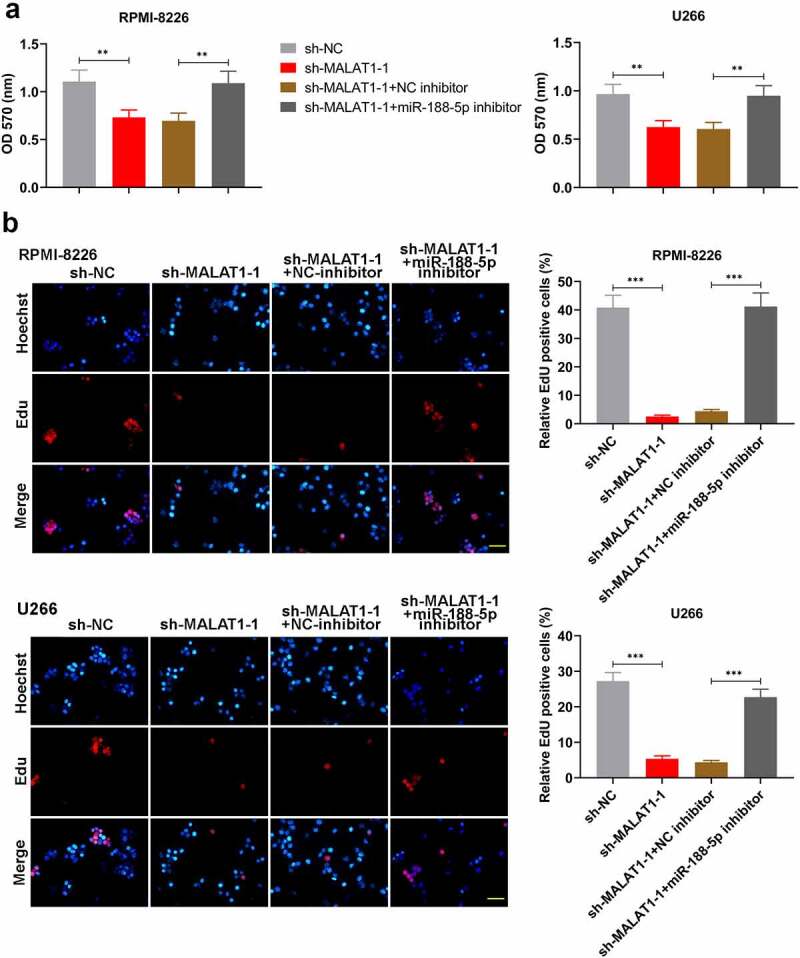
MiR-188-5p knockdown restores the inhibition of cell proliferation in MALAT1-slilenced MM cells. (a). MTT assay was performed to measure the cell viability in RPMI-8226 and U266 cells 48 h after co-transfection. (b). EdU staining (red) was performed to check proliferating cells 48 h after co-transfection. Cells were counter-stained by Hoechst 33,342 (blue). Scale bar = 50 μm. **P < 0.01, ***P < 0.001

**Figure 8.**
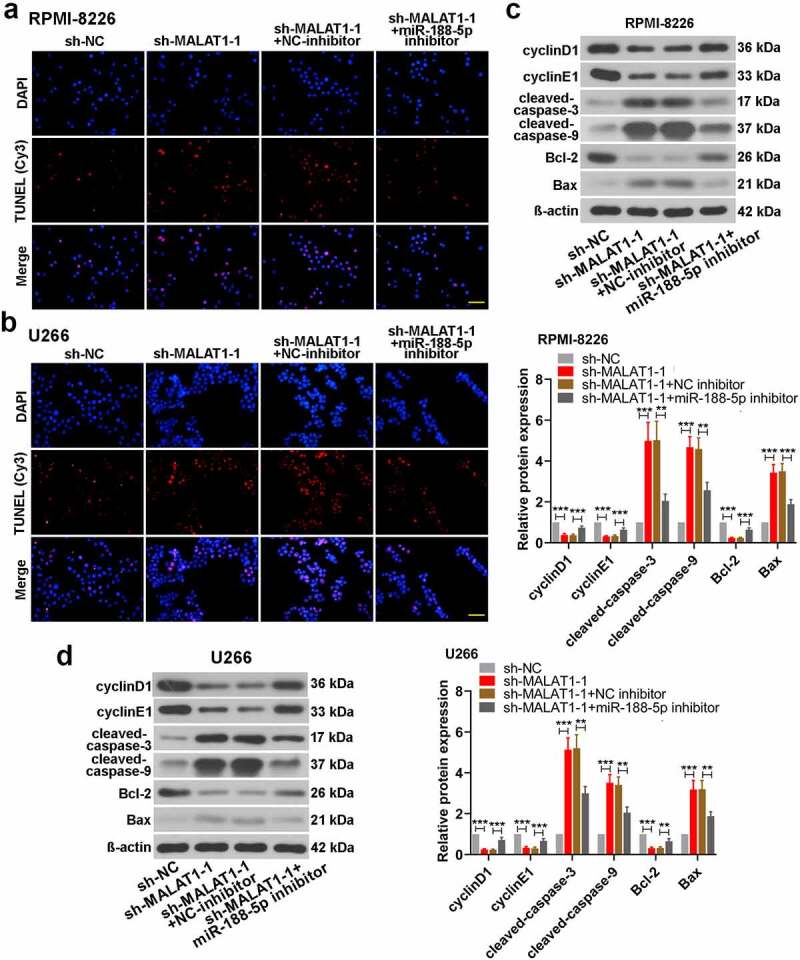
MiR-188-5p knockdown relieves cell apoptosis induced by sh-MALAT1-1 in the MM cells. (a and b). TUNEL staining (Cy3, red) was performed to detect the apoptotic cells 48 h after co-transfection in the RPMI-8226 and U266 cells combined with DAPI staining (blue). Scale bar = 50 μm. (c and d). WB analysis of cell cycle- and apoptosis-related proteins in MM cell lines 48 h after co-transfection. **P < 0.01, ***P < 0.001

To further investigate whether miR-188-5p was involved in MALAT1-triggered regulation of MM cell development, miR-188-5p inhibitor and sh-MALAT1-1 were co-transfected into the RPMI-8226 and U266 cells. MTT assay and Edu staining were performed to measure the cell viability and proliferating cells at 48 h after co-transfection, respectively. On the basis of MALAT1 knockdown, miR-188-5p inhibitor could obviously restore the reductions in the viability of the MM cells (Figure 7(a)). Similarly, further miR-188-5p downregulation also reversed the sh-MALAT1-1-induced decreases in the number of EdU-positive cells and led to DNA replication recovery (Figure 7(b)). On the other hand, apoptosis analysis was carried out through TUNEL staining, which showed that miR-188-5p knockdown inhibited the sh-MALAT1-1-induced cell apoptosis in the MM cells (Figure 8(a and b)). The results of WB analysis also verified these alterations which showed that further miR-188-5p inhibition could restore the inhibitions in the expression of cyclinD1, cyclinE1 and Bcl-2 and the promotions in the expression of cleaved caspase-3 and 9 and Bax in the MM cells induced by MALAT1 knockdown (Figure 8(c and d)). It was indicated that miR-188-5p knockdown recovered the decreased cell proliferation and alleviated the enhanced cell apoptosis induced by downregulated MALAT1. These results collectively suggest that miR-188-5p is involved in MM cell progression regulated by MALAT1.

## Discussion

Since previous study has demonstrated that downregulation of lncRNA MALAT1 suppressed cell proliferation and induced apoptosis in the MM [20], further molecu les involved in the functions of MALAT1 in MM development aroused our interests. Two binding sites of miR-188-5p were found in MALAT1 using online prediction tool, which brought a proper hypothesis that miR-188-5p might mediate the functions of MALAT1 in MM tumor growth. Although miR-188-5p has been manifested as a tumor suppressor in many cancers, like gastric cancer, hepatocellular carcinoma, prostate cancer, glioma and nasopharyngeal cancer [14–18], the study on it in MM tumors has not explored. In the present work, functional analysis indicated that miR-188-5p inhibited cell proliferation and enhanced cell apoptosis in the MM in vitro and in vivo. It was also proved that MALAT1 directly targeted miR-188-5p to regulate its expression levels and miR-188-5p was involved in the functions of MALAT1 in MM progression. Both MALAT1 and miR-188-5p work together to modulate MM tumor development. Although further downstream pathways or molecules of miR-188-5p in the MM have not been investigated in this study, it at least provided evidences that MM tumor development could be altered through exogenous regulation of miR-188-5p which may be considered for MM treatment.

However, some previous studies have demonstrated several downstream targets of miR-188-5p in other cancers. For example, it was found that β-catenin was a direct target of miR-188, which could induce the activation of Wnt/β-catenin pathway to regulate cell proliferation in the glioma [17]. Besides, a fibroblast growth factor, FGF5 was also found to be targeted on miR-188-5p directly and contributed to the hepatocellular cancer progression [15]. Moreover, miR-188-5p was shown to regulate PI3K/AKT signaling through directly targeting lysosomal protein transmembrane protein 4B, which was associated with prostate cancer cell development [16]. Regardless in which cancer type, the pathological functions of all these targets were closely linked to cell proliferation, tumorigenesis and tumor growth [21–24]. As miR-188-5p was demonstrated to be inversely correlated with these oncogenic targets, it mainly functioned as a tumor suppressor which was also consistent with the findings in present study.

From the WB results here in vitro and in vivo, overexpression of miR-188-5p in the MM cells decreased the protein levels of cyclinD1 and cyclinE1, indicating a suppressed G1/S transition during cell cycle [25]. A previous research has demonstrated that miR-188 directly bound CCND1 and CCNE1 that encoded cyclinD1 and cyclinE1 to inhibit cell cycle progression in the CNE cells [18], which also provides evidences to support present results in the MM cells. According to the interaction of RB with E2F, phosphorylated Rb can be released from Rb/E2F proteins and then activates E2F activities to initiate transcription and further gene expressions [18,26]. However, miR-188-5p upregulation here disturbed this Rb/E2F interaction, resulting from remarkably decreased phosphorylation of Rb mediated by downregulated cyclins. Finally, it caused suppressed DNA replication and cell proliferation in the MM cells. Additionally, increased apoptosis was found in the miR-188-5p overexpressed MM cells as evidenced by elevated cleaved-caspase-3 and 9, reduced pro-survival protein Bcl-2 and enhanced pro-apoptotic protein Bax, indicating an activation of endogenous apoptosis pathways [27] in the MM cells. Therefore, exogenous dysregulation of miR-188-5p in the MM cells triggered both cyclin/Rb/E2F-related signaling and Bcl-2/Bax/caspase-related pathway to modulate cell fate during tumor development. The downstream targets or molecules of miR-188-5p in the MM cells also need more time to investigate.

Notably, the effects of MM tumor progression induced by MALAT1 knockdown [20] were similar with those induced by miR-188-5p upregulation in the present study. Besides, it was shown that MALAT1 negatively regulated miR-188-5p expressions via directly binding to miR-188-5p and miR-188-5p was also engaged in the regulations of MM cell fate by MALAT1. Taken together, it was possible that MALAT1 might sponge miR-188-5p through one or two MREs to affect expressions of its target gene(s) in the MM cells. However, there were still some open questions that need to be taken into consideration in our future study, like which downstream target gene(s) of MALAT1/miR-188-5p to affect MM cell activities, whether MALAT1 contains different MREs of other miRNAs and also whether miR-188-5p shares MREs with other transcriptome.

## Conclusion

In conclusion, our present study demonstrates that overexpression of miR-188-5p significantly decreases MM cell proliferation but enhances cell apoptosis of the MM in vitro and in vivo. MALAT1 is indicated to negatively regulate miR-188-5p and miR-188-5p is shown to mediate the function of MALAT1 in MM cell development through directly binding to MALAT1. These findings not only provide a new cancer type, the MM, to the regulatory networks of tumor suppressor miR-188-5p, but also afford evidences to regard miR-188-5p as a potential therapeutic target or prognostic indicator for future MM treatment.

## Supplementary Material

Supplemental Material

## Data Availability

The datasets used and/or analyzed during the current study are available from the corresponding author on reasonable request.

## Highlights

1. MiR-188-5p inhibits multiple myeloma (MM) cell proliferation.

2. MiR-188-5p enhances MM cell apoptosis.

3. MiR-188-5p suppresses MM tumor growth.

4. LncRNA MALAT1 bound to miR-188-5p to negatively regulate miR-188-5p.

5. LncRNA MALAT1 regulated MM cell proliferation and apoptosis via miR-188-5p.
