# Supplementary material for: MicroRNA miR-188-5p as a mediator of long non-coding RNA MALAT1 regulates cell proliferation and apoptosis in multiple myeloma
Source: Bioengineered. 2021 May 4;12(1):1611–26. doi: 10.1080/21655979.2021.1920325 (PMC8806342; doi:10.1080/21655979.2021.1920325)
Supplement: Supplemental Material [file KBIE_A_1920325_SM3541.zip › Document.rtf]

Supplementary materials
Figure S1 Effect of miR-188-5p on the viability of U266 and NCI-H929 cells. U266 cells were transfected with miR-188-5p inhibitor, while NCI-H929 cells with miR-188-5p mimic. A and B. Cell viability at 0, 24, 48, 72, 96 h after transfection was detected using MTT assay. *P < 0.05 and **P < 0.01 compared with the NC mimic or inhibitor group.
Figure S2 Pearson correlation analysis between MALAT1 and miR-188-5p expressions in MM cells. 
