# Supplementary figures and images for: MicroRNA miR-188-5p as a mediator of long non-coding RNA MALAT1 regulates cell proliferation and apoptosis in multiple myeloma
Source: Bioengineered. 2021 May 4;12(1):1611–26. doi: 10.1080/21655979.2021.1920325 (PMC8806342; doi:10.1080/21655979.2021.1920325)

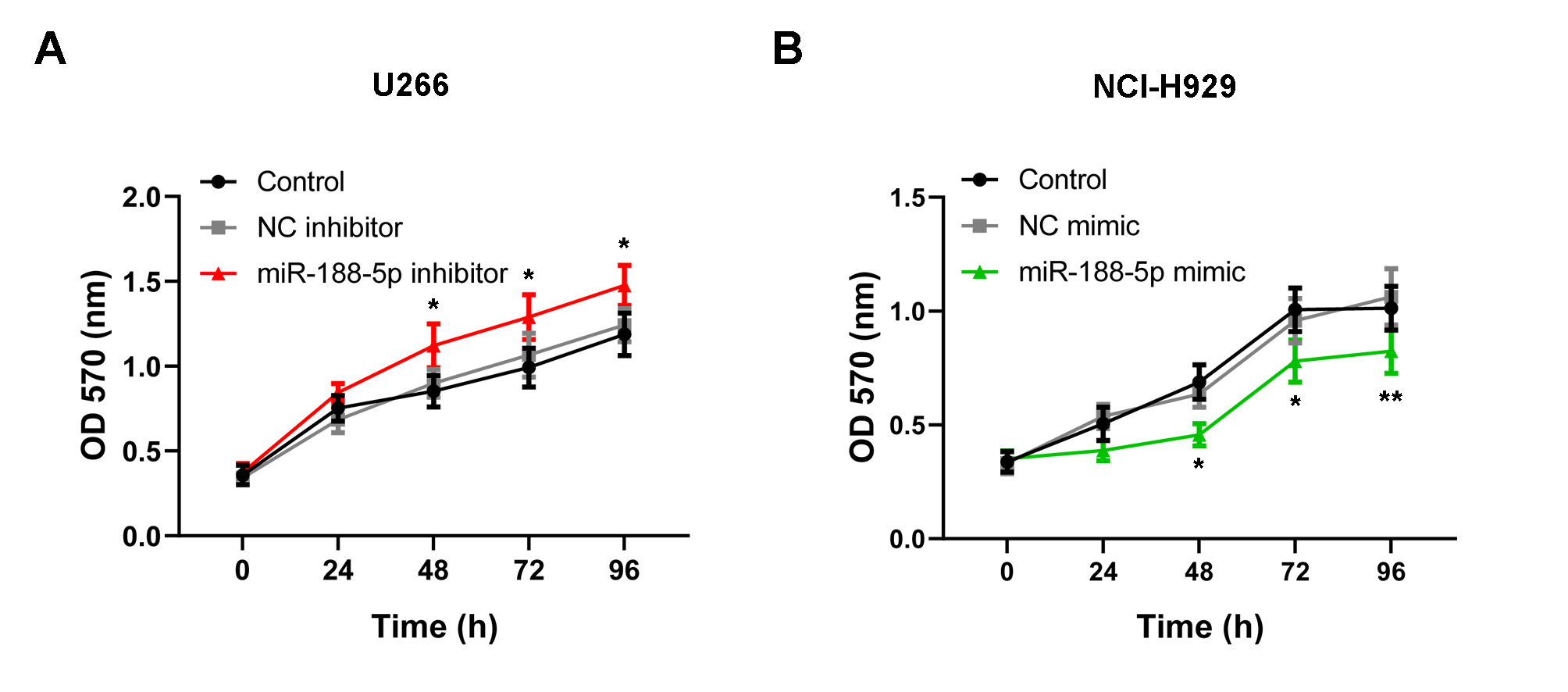

Supplement: Supplemental Material [file KBIE_A_1920325_SM3541.zip › Figure S1.tif]

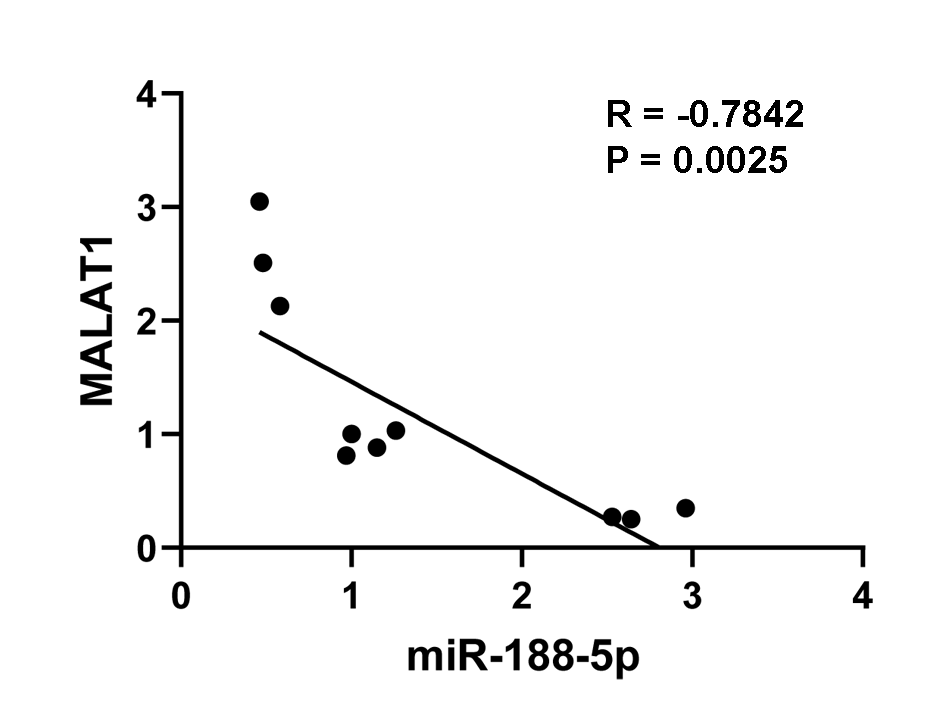

Supplement: Supplemental Material [file KBIE_A_1920325_SM3541.zip › Figure S2.tif]
